# Inflammatory profile associated with hyperglycemia in children with type 1 diabetes

**DOI:** 10.1016/j.jdiacomp.2026.109275

**Published:** 2026-02-01

**Authors:** Nicole Glaser, Zachary Chaffin, Daniel Tancredi, Arleta Rewers, Marian Rewers, Spencer Gilles, Bradley Ander, Simona Ghetti

**Affiliations:** a Section of Endocrinology, Department of Pediatrics, University of California Davis School of Medicine, Sacramento, CA, USA; b Section of Critical Care Medicine, Department of Pediatrics, University of California Davis School of Medicine, Sacramento, CA, USA; c Section of Emergency Medicine, Department of Pediatrics, University of Colorado School of Medicine, CO, USA; d Barbara Davis Center for Diabetes, University of Colorado School of Medicine, CO, USA; e Department of Neurology, University of California Davis School of Medicine, Sacramento, CA, USA; f Department of Psychology, University of California Davis, Davis, CA, USA

**Keywords:** Type 1 diabetes, Children, Inflammation, Cytokines, Hyperglycemia

## Abstract

**Background:**

Complications of type 1 diabetes (T1D) are associated with exposure to hyperglycemia. Inflammation is involved in microvascular and macrovascular complications, but associations between hyperglycemia and inflammatory mediators across multiple classes have not been comprehensively described. We aimed to characterize the inflammatory profile associated with hyperglycemia in children with T1D.

**Methods:**

We comprehensively evaluated blood inflammatory mediators (cytokines, chemokines, growth factors, matrix metalloproteinases (MMPs)) using multiplex immunoassays in 117 children with T1D. We used multiple linear regression analyses to assess the relations between inflammatory mediators (transformed to robust z-scores) and hemoglobin A1c (HbA1c), adjusting for age, diabetes duration and body mass index. We computed an inflammatory composite as the within-person mean of significantly associated robust z-scores.

**Results:**

Levels of multiple inflammatory mediators were associated with HbA1c (*p* < 0.05 and False Discovery Rate-adjusted q < 0.10). These included *cytokines* [interleukin (IL)-1β, IL-1 receptor antagonist, IL-2, IL-4, IL-6, IL-8, IL-13, IL-17A, IL-17F, IL-18, IL-20, IL-21, IL-23, IL-33, interferon-γ, tumor necrosis factor (TNF)-α, TNF-related apoptosis inducing ligand (TRAIL), thrombopoietin, stem cell factor, leukemia inhibitory factor, *chemokines* (CCL1, CCL2, CCL4, CCL7, CCL8, CCL13, CCL26, CCL27, CXCL1, CXCL5, CXCL13), *growth factors* (vascular endothelial growth factor-A, transforming growth factor-α, platelet derived growth factor-AA), and *MMPs* (MMP-1, MMP-2, tissue inhibitor of MMP-1). Each percentage point increase in HbA1c was associated with a 0.16 increase in inflammatory composite score.

**Conclusions:**

A broad range of inflammatory mediators are correlated with HbA1c in children with T1D. These inflammatory changes precede development of T1D complications, suggesting that possible pathophysiologic involvement should be investigated.

## 1. Introduction

Inflammation is thought to play a pathogenic role in complications of type 1 diabetes (T1D).^1–5^ Alterations in levels of various inflammatory biomarkers are associated with risk and progression of both microvascular and macrovascular complications of T1D.^2^ Greater exposure to hyperglycemia is strongly associated with increased risk of T1D complications,^6,7^ and hyperglycemia has been found to increase levels of some inflammatory mediators. However, only limited numbers of mediators have been studied in people with T1D and the inflammatory state resulting from hyperglycemia has not been comprehensively described.

The pathogenesis of T1D complications involves a complex interplay among multiple factors including inflammation, oxidative stress, endothelial injury and hemodynamic changes.^2^ Furthermore, inflammatory changes may both cause and result from vascular and end-organ damage making causal relationships difficult to elucidate.^1,5,8^ A wider range of inflammatory mediators has been studied in adults with T1D than in children with T1D, however, studies in children may be particularly informative. T1D complications are infrequent in this age group, allowing for characterization of the inflammatory state in the absence of substantial changes resulting from vascular or end-organ damage. Previous studies of children with T1D have investigated individual inflammatory mediators or small groups of mediators,^9–11^ however, to our knowledge no single study has extensively characterized the relations between hyperglycemia and inflammatory mediators across multiple inflammatory class types in children with T1D. Characterization of inflammatory patterns resulting from T1D in childhood is particularly important such that associations of these patterns with long-term complications can be determined. Previous studies have particularly identified Il-1β, IL-6, IL-18, C-reactive protein, and TNF-α as important mediators of inflammation resulting from hyperglycemia.^12–15^ Altered levels of other inflammatory mediators, particularly chemokines and growth factors, have been identified in people with microvascular complications of T1D.^2,16^ but it is unclear whether these alterations are a cause or a consequence of end-organ damage. We hypothesized that the inflammatory profile of hyperglycemia would extend beyond the group of inflammatory markers known to be elevated in uncomplicated T1D and would include a diverse array of inflammatory mediators. In the current study, we assessed a comprehensive panel of inflammatory mediators in children with T1D and determined associations of these mediators with HbA1c.

## 2. Methods

### 2.1. Study population

This study was approved by the institutional IRB at the participating institutions. The study analyzed blood samples from children with type 1 diabetes (T1D) collected at two clinical sites, UC Davis Children’s Hospital and the Barbara Davis Center for Diabetes. Blood Samples from UC Davis Children’s Hospital were obtained from children attending diabetes clinic visits during which phlebotomy was planned for routine care (*n* = 44). Samples from the Barbara Davis Center for Diabetes were obtained from youth participating in a cohort study of predictors of early cardiovascular disease (*n* = 73).^17^

Samples were obtained from patients 18 years or younger who were diagnosed with T1D and had duration of diabetes of one year or longer. Prospectively enrolled patients at UC Davis Children’s Hospital were excluded if they had fever or other signs of infection, had been diagnosed with an infectious illness within the two weeks preceding enrollment or had an episode of DKA within the 2 months prior to the visit. Patients were excluded if they had autoimmune thyroid disease with abnormal thyroid function studies. Patients with positive thyroid antibodies but with normal thyroid function studies were included, as were patients treated for hypothyroidism with levothyroxine who had normal thyroid function studies. Patients with untreated celiac disease or any other autoimmune or inflammatory condition were excluded, Patients were also excluded if they had microalbuminuria or had been diagnosed with diabetic kidney disease or other microvascular complications of diabetes. We recorded HbA1c levels measured for routine clinical care on the day of blood sample collection. Additional HbA1c levels measured since the time of diagnosis of T1D were also recorded from the medical record.

Children who contributed blood samples to the biobank at the Barbara Davis Center for Diabetes were not acutely ill at the time of sample collection and had not been diagnosed with hypothyroidism or hyper-thyroidism. None of the participants contributing samples to the biobank had microalbuminuria, diabetic kidney disease or other microvascular complications of diabetes. Children contributing samples to the biobank had not had DKA episodes within the preceding 3 months. Samples from children with celiac disease following gluten free diets (*n* = 3) were included. No samples were obtained from children with untreated celiac disease.

### 2.2. Sample processing and analysis

Blood samples from clinic patients were collected in 5 mL plasma EDTA tubes and gently inverted 10–20 times. Tubes were then centrifuged at 1473 RCF for 15 min. Plasma samples were divided into 5 aliquots tubes and stored at −80 °C until the time of shipping. We measured a comprehensive panel of 84 inflammatory mediators including cytokines, chemokines, growth factors, matrix metalloproteinases (MMP) and tissue inhibitors of matrix metalloproteinases (TIMPs). Both prospectively collected samples and biobank samples were shipped to a CLIA certified lab (Eve Technologies Corp., Calgary, Alberta) for analysis. Multiplexed immunoassays were employed for these measurements using Luminex xMAP technology on the LuminexTM 200 system (Luminex, Austin, TX, USA). Cytokines, chemokines, and growth factors were simultaneously measured in the samples using several multiplex kits (HCYTA-60 K-PX48 and HCP2MAG-62 K-PX23 from Millipore Sigma, Burlington, Massachusetts, USA, and #FCSTM07/MMP and #LKTM003/TIMP from R&D Systems, Inc., Minneapolis, MN, USA). All assays were run according to the manufacturer’s protocol with separate runs containing a consistent reference sample to monitor for any technical drift.

### 2.3. Statistical analysis

Inflammatory mediator levels were natural log-transformed to approximate a normal distribution. To account for possible variability among assays (i.e. batch effects) as well as for ease of comparison among inflammatory mediators, we calculated batch-specific robust z-scores for each mediator, subtracting the median values from children with glycemic levels at or near target (reference group) to generate a deviation and then dividing these deviations by a robust measure of scale based on the residuals from a regression model of these deviations that include fixed effects for batch. *Z*-scores were winsorized to lie between −4 and 4 to lessen the influence of skewed outliers.

Associations between inflammatory mediator z-scores and HbA1c were evaluated using linear regression models. Covariates that might affect inflammatory marker levels including age, duration of diabetes, and body mass index (BMI)^18,19^ were included in the regression model. To account for repeated comparisons, we used the two-stage Benjamini, Yekutieli & Krieger approach to compute adjusted *p*-values to control the false discovery rate at 10% for the family of regression-adjusted comparisons pooled across all of the mediators examined as outcomes.^20^

We created an inflammatory composite z-score by calculating the mean z-score of all inflammatory mediators that were found to be significantly associated with HbA1c in individual analyses. *Z*-scores that were inversely associated with HbA1c were included in the equation as the negative of the calculated z-score. HbA1c at the time of sample collection and mean HbA1c since diagnosis were compared using Student’s *t*-test. All statistical analyses were performed using Stata version 17.0.

## 3. Results

### 3.1. Population description

Demographic data for the 117 study participants is presented in Table 1. HbA1c at the time of blood sample collection ranged from 6.1 to 14% with a mean of 8.8%. For 39 study participants, all HbA1c values measured since diagnosis were accessible in the medical records. HbA1c measured at the time of sample collection for these participants (8.3 ± 1.7%) was similar to the mean HbA1c since diagnosis (8.3 ± 1.5%, *p* = 0.63) suggesting that HbA1c tended to be stable over time within individuals.

### 3.2. Inflammatory mediators

**Fig. 1.**
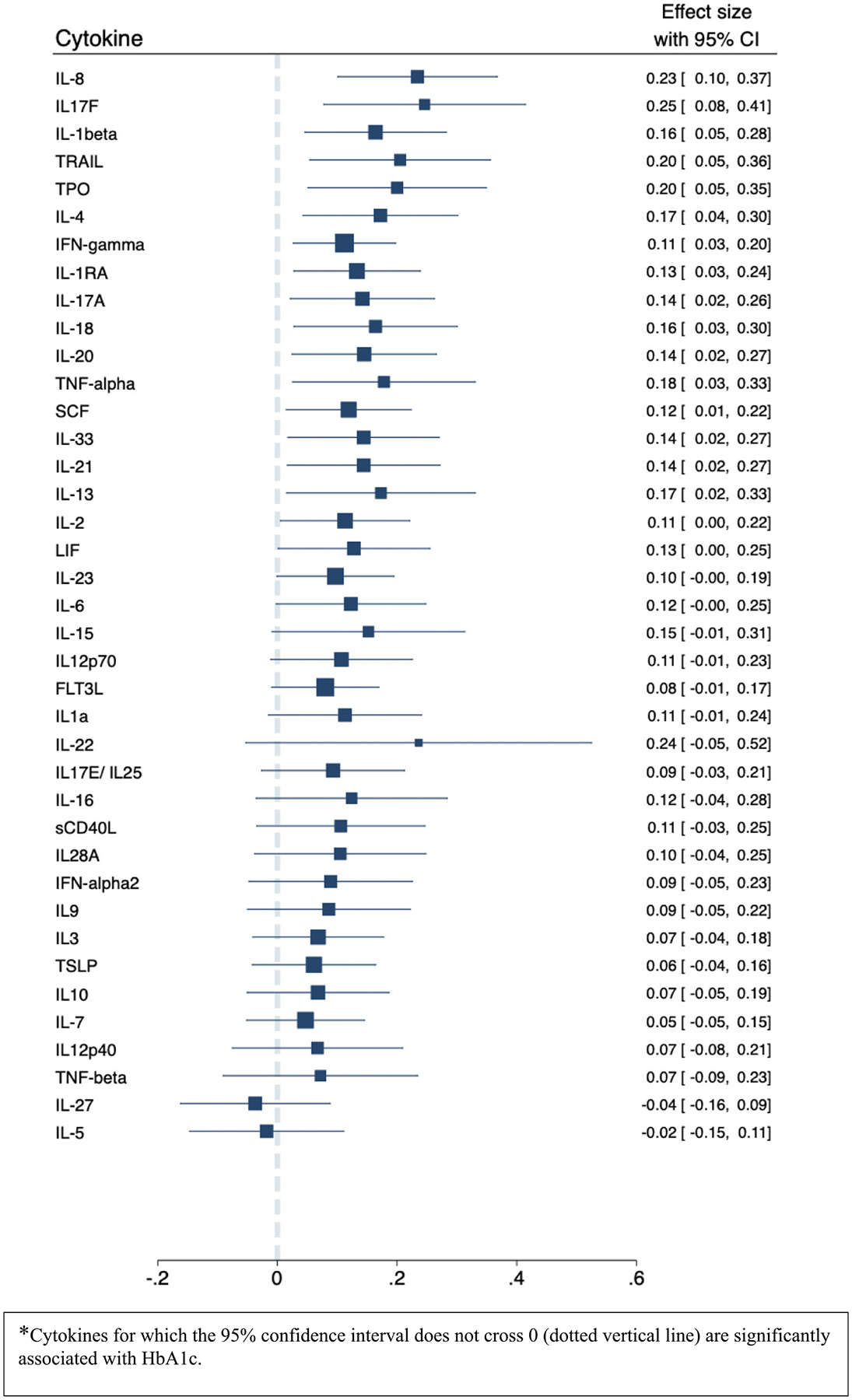
Associations of cytokine z-scores with HbA1c. Effect sizes report the estimated increase in cytokine z-score for each 1% increase in HbA1c, adjusting for age, duration of diabetes and BMI. *Cytokines for which the 95% confidence interval (CI) does not cross zero are significantly associated with HbA1c (p < 0.05 and False Discovery Rate-adjusted q < 0.10)*. Results for GM-CSF are not shown due to high frequency of undetectable levels (>75%) in one or more assay batches.

**Fig. 2.**
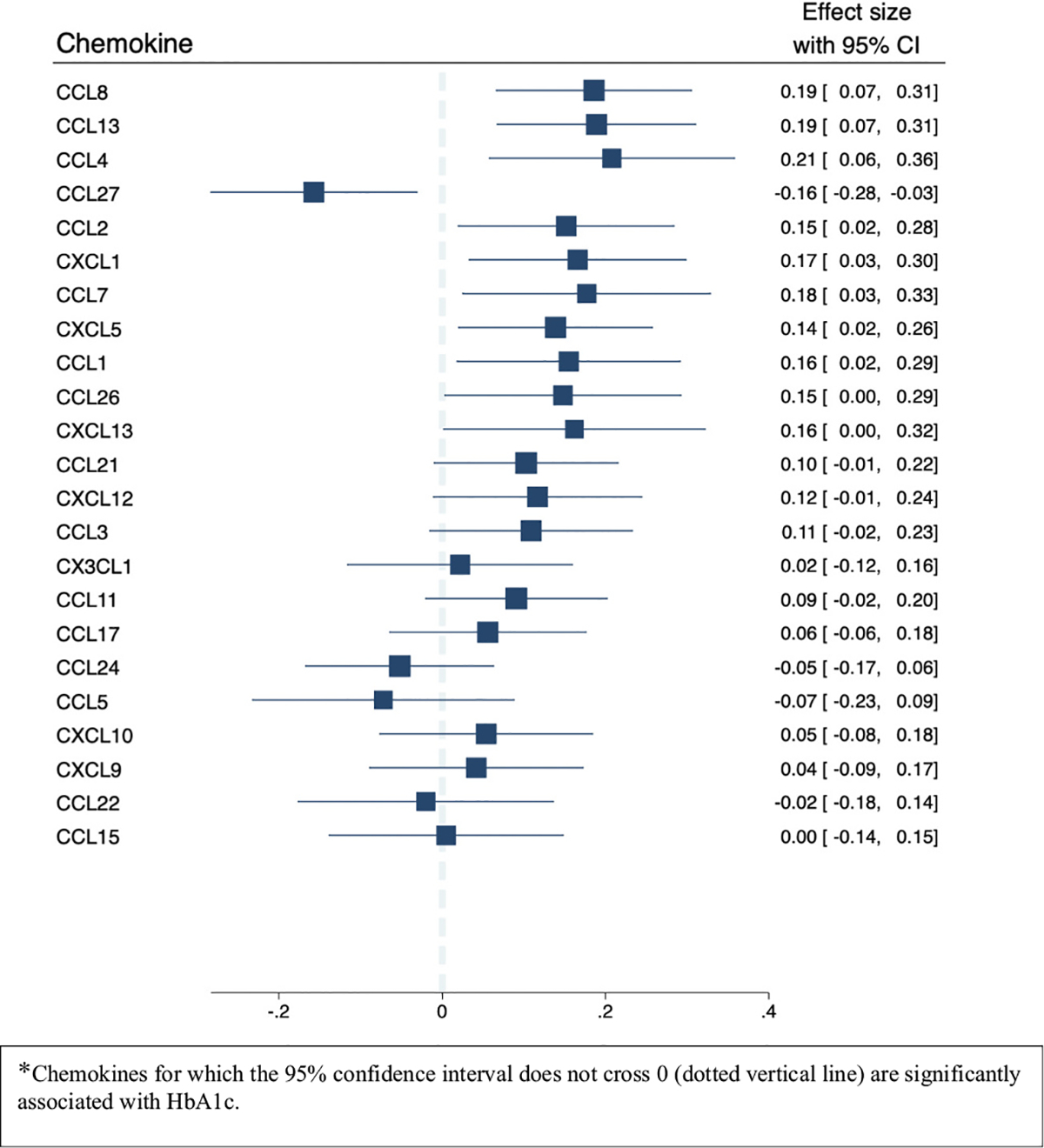
Associations of chemokine z-scores with HbA1c. Effect sizes report the estimated increase in chemokine z-score for each 1% increase in HbA1c, adjusting for age, duration of diabetes and BMI. Chemokines *for which the 95% confidence interval (CI) does not cross zero are significantly associated with HbA1c (p < 0.05 and False Discovery Rate-adjusted q < 0.10)*.

**Fig. 3.**
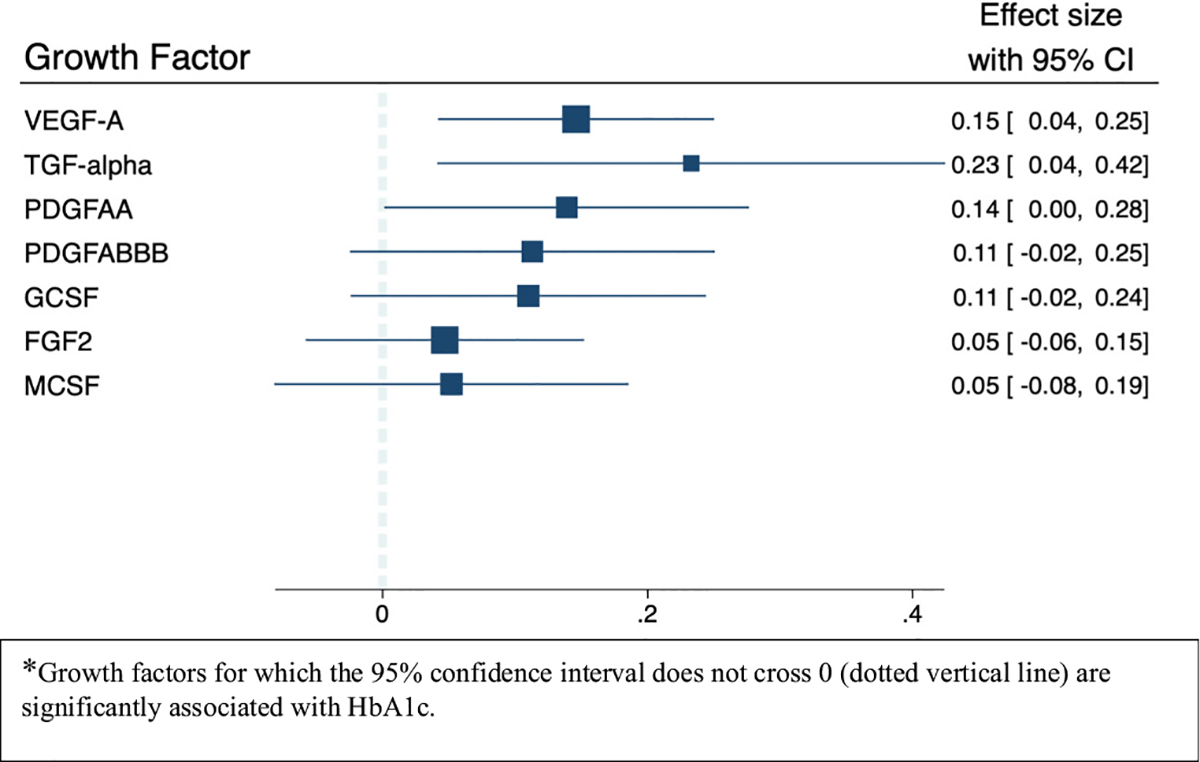
Associations of growth factor z-scores with HbA1c. Effect sizes report the estimated increase in growth factor z-score for each 1% increase in HbA1c, adjusting for age, duration of diabetes and BMI. Growth factors *for which the 95% confidence interval (CI) does not cross zero are significantly associated with HbA1c (p* < *0.05 and False Discovery Rate-adjusted q* < *0.10)*. Results for Epidermal Growth Factor (EGF) are not shown due to high frequency of undetectable levels (>75%) in one or more assay batches.

**Fig. 4.**
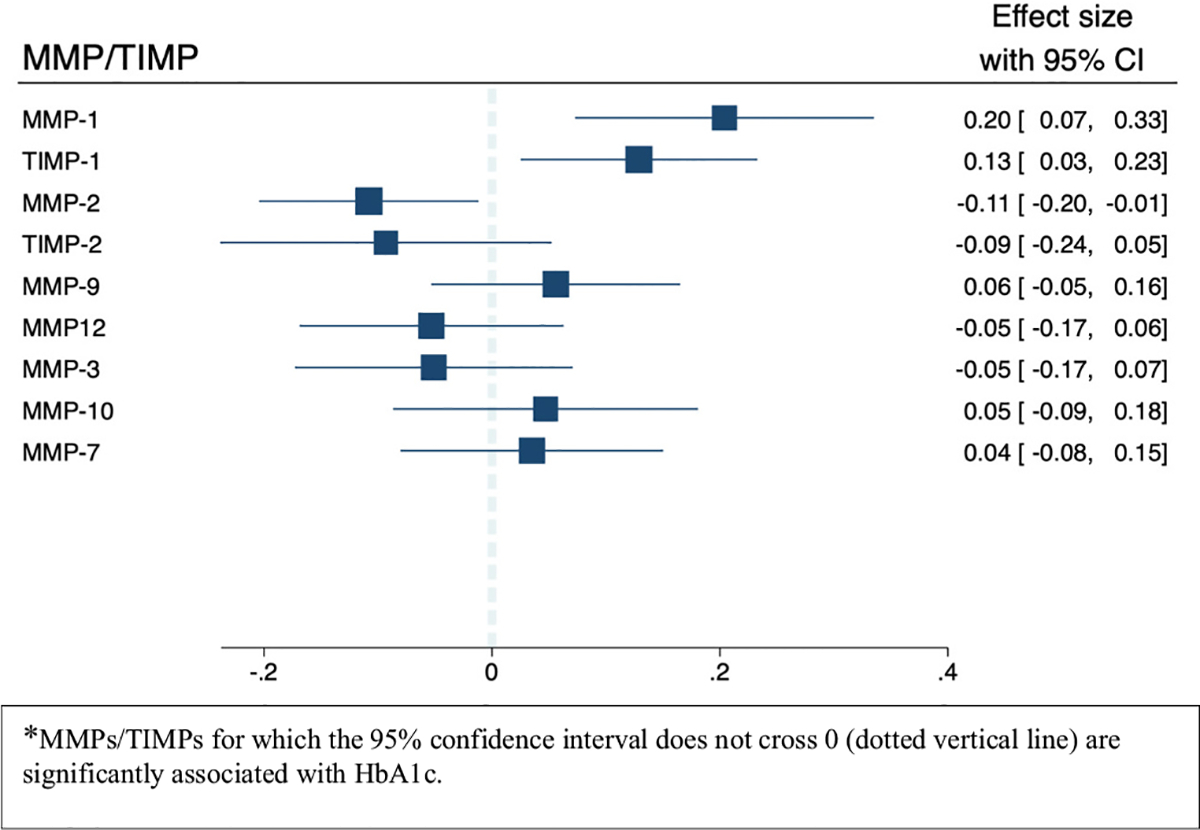
Associations of MMP/TIMP z-scores with HbA1c. Effect sizes report the estimated increase in MMP/TIMP z-score for each 1% increase in HbA1c, adjusting for age, duration of diabetes and BMI. MMP/TIMPs *for which the 95% confidence interval (CI) does not cross zero are significantly associated with HbA1c (p* < *0.05 and False Discovery Rate-adjusted q* < *0.10)*. Results for MMP-8, MMP-13 and TIMP3 are not shown due to high frequency of undetectable levels (>75%) in one or more assay batches.

In linear regression models adjusting for age, duration of diabetes and BMI, we found that levels of multiple inflammatory mediators were significantly associated with HbA1c. Among the 40 cytokines measured, 20 showed significant associations with HbA1c after correcting for the FDR. The strongest associations were noted for IL-8, IL-17F, IL-1β, tumor necrosis factor related apoptosis inducing ligand (TRAIL), and thrombopoietin (TPO, Fig. 1). Among the 23 chemokines measured, 11 were significantly associated with HbA1c with CCL8, CCL13 and CCL4 showing the strongest associations (Fig. 2). Several growth factors (VEGF-A, TGFα and PDGFAA, Fig. 3) and MMPs (MMP1, TIMP1 and MMP2, Fig. 4) were also significantly correlated with HbA1c level.

### 3.3. Inflammatory composite score

**Fig. 5.**
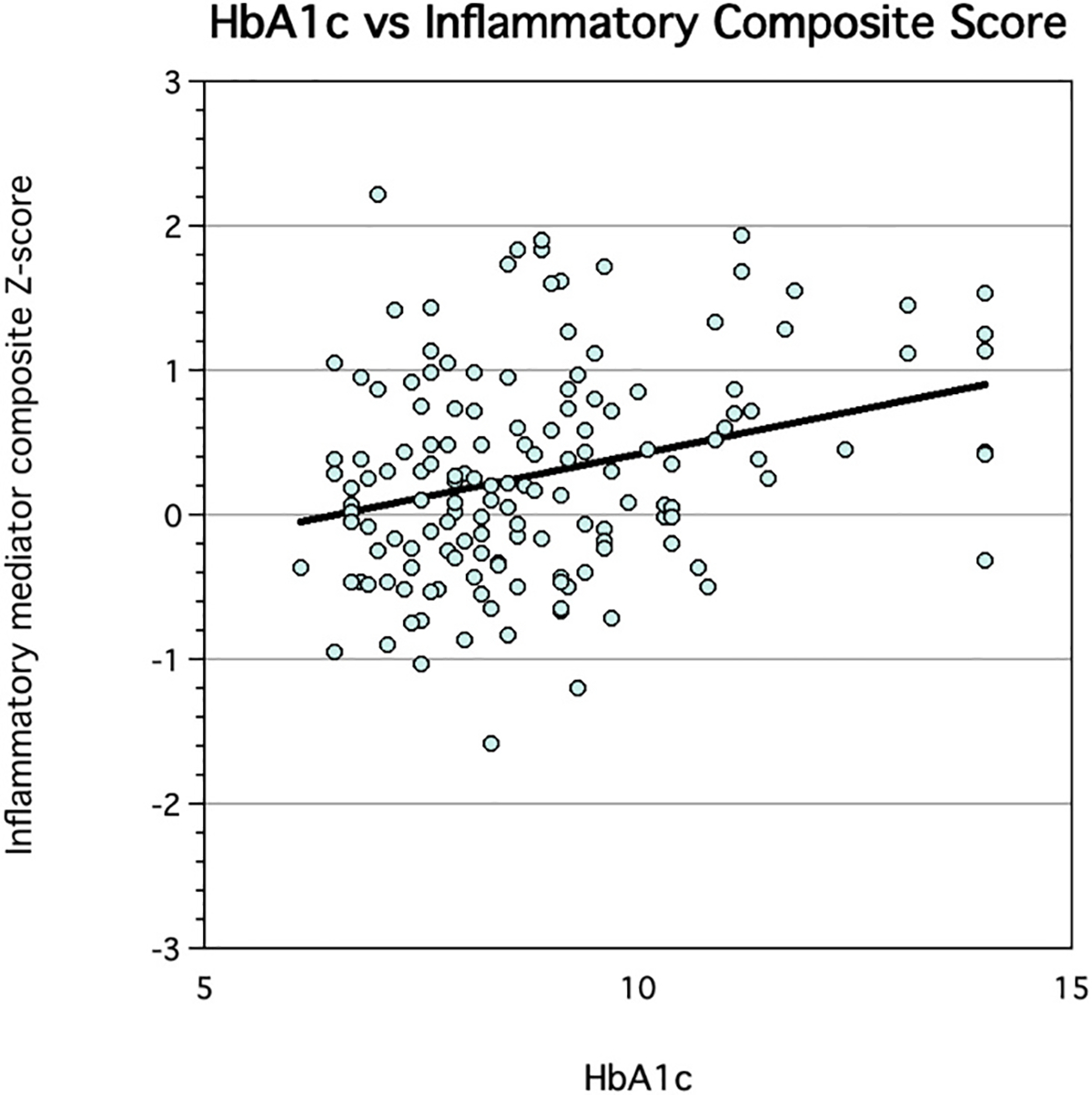
Correlation of HbA1c with inflammatory mediator composite scores. z-scores for all inflammatory mediators significantly associated with HbA1c (see Figs. 1–4) were averaged to create an inflammatory composite score. Inflammatory mediators that were inversely correlated with HbA1c were included as the negative of the z-score.

The inflammatory composite score, calculated as the mean z-score of all inflammatory mediators that were significantly associated with HbA1c in individual analyses, was strongly associated with HbA1c in a model adjusting for age, duration of diabetes and BMI (Fig. 5). Results from the regression model suggest that each 1 percentage point increase in HbA1c is associated with an increase in the mean inflammatory composite score of 0.16 (95% CI 0.09, 0.23, *p* < 0.001).

## 4. Discussion

Substantial evidence demonstrates that hyperglycemia is the driving factor leading to complications of T1D.^6,7^ However, the pathobiology linking hyperglycemia to organ damage is complex and incompletely understood. Inflammation is thought to play a role in causing vascular and end organ damage, with elevated circulating and organ-specific levels of inflammatory mediators documented in diabetic kidney disease (DKD), diabetic retinopathy and diabetic neuropathy.^2^ However, many studies of inflammation in patients with diabetes involved adult participants and included individuals with type 2 diabetes, a disorder in which additional metabolic factors (obesity, insulin resistance and hyperlipidemia) are known to increase systemic inflammation.^21^ Furthermore, whether elevated inflammatory levels are a cause or a consequence of organ damage is difficult to distinguish in patients with established complications of diabetes. Measuring inflammatory mediators in children with T1D, prior to the occurrence of complications, therefore provides important insights into the roles of inflammatory pathways in contributing to complications of diabetes. In addition, previous studies of inflammation in children with T1D have investigated limited numbers of inflammatory markers such that the inflammatory profile of hyperglycemia in T1D has been incompletely characterized. In the current study, we comprehensively characterized the inflammatory profile across multiple inflammatory mediator classes in children with T1D. We documented that levels of multiple cytokines, chemokines, growth factors and matrix metalloproteinases are significantly correlated with HbA1c. Although multiple factors can affect systemic inflammation, we demonstrate that the inflammatory signal resulting from hyperglycemia is readily evident in children with T1D after adjusting for age, diabetes duration and BMI.

To our knowledge, no previous study has broadly characterized the inflammatory profile associated with hyperglycemia in children with T1D. However, several studies have investigated limited numbers of inflammatory markers in this population. Similar to our findings, VEGF levels have been shown to correlate with glycemia and are increased in youth with microvascular complications of T1D.^22^ Additional studies have found elevated levels of IL-6, IL-18, TNA-α, IL-18, CCL2, CXCL1, PDGF-BB, PDGF-AA, sCD40L MMP-9,TIMP-1, fibrinogen and epidermal growth factor in adolescents with T1D compared to healthy controls.^10–12,23^ Increased mRNA expression of IL-1β, IL-6, IL-18, TNF-α, and CCL2 has also been documented in youth with T1D compared to healthy controls.^24,25^ In adults with T1D, IL-6 levels have been found to be significantly elevated in patients with HbA1c above 7%.^14^ However, studies using continuous glucose monitoring (CGM) found that IL-6 levels were positively correlated with time below range in the previous 14 days and negatively correlated with time above range, suggesting that more recent versus longer-term glycemic changes may have different effects on inflammation.^26^

Comparisons between studies investigating inflammatory mediators in patients with established microvascular complications of T1D and the current study are potentially informative in highlighting which inflammatory factors might play a role in the early stages of organ injury and which occur at later stages or result from organ injury. In adults, circulating levels of various inflammatory mediators are associated with microvascular and macrovascular complications of T1D.^2^ Among inflammatory mediators found to be significantly correlated with HbA1c in the current study, several are known to be serum biomarkers of DKD in adults. These include IL-1β, IL-1RA, IL-2, IL-6, IL-17F, IFN-γ, TNF-α, VEGF, CCL2, CCL4, MMP-1 and TIMP-1.^8,27–29^ Other serum biomarkers of DKD, including TNF-β, sCD40, CCL11,CCL15, CCL3, MMP-3, MMP-9 and MMP-10 were not significantly associated with HbA1c levels in the current study.^30^ Interestingly, although MMP-2 levels were negatively correlated with HbA1c in the current study, elevated MMP-2 levels are associated with DKD in adults.^31^ Levels of IL-1β, IL-6, IL-8, TNF-α, CCL2 and VEGF in the vitreous humor are also associated with diabetic retinopathy in T1D.^32^ As in DKD, increased circulating levels of MMP-2 have been found to be associated with increased severity of diabetic retinopathy.^30,31^ Our findings suggest that increases in MMP-2 as well as TNF-β, sCD40, CCL11,CCL3, MMP-3, MMP-9 and MMP-10 may occur at later stages in the development of diabetes-related complications or might result from complications, rather than contributing to the initial pathogenesis.

Multiple inflammatory markers are also associated with diabetic neuropathy. Elevated levels of IL-6, IL-18 and IL-1RA have been found to be associated with peripheral neuropathy in older adults with type 2 diabetes.^33,34^ In adults with T1D, elevated circulating levels of IL-4, IL-1α and CCL-2 are associated with autonomic neuropathy^35^ and elevated levels of TNF-α and CXCL9 with peripheral neuropathy.^36–38^ In a study including adults with either T1D or T2D, higher circulating levels of PDGF AA/BB, CCL5, G-CSF, TNFα were found in patients with neuropathy compared to those without.^39^ Apart from CXCL9, IL-1α and G-CSF (and PDGF AA/BB, which was not measured) all these inflammatory mediators were significantly associated with HbA1c in the current study.

Associations between inflammatory biomarkers and macrovascular complications of diabetes have also been documented. Elevated circulating levels of IL-1β, IL-1RA, IL-6, IL-8, TNF-α, sCD40, VEGF, CXCL10, CCL2 and TIMP-1 are associated with cardiovascular disease in studies of adults with diabetes.^30,40,41^ Apart from sCD40 and CXCL10, all these inflammatory mediators were significantly correlated with HbA1c in the current study.

In addition to having possible causal links to T1D complications, chronic inflammation in T1D may also have relevance for other disorders that occur with increased frequency in individuals with T1D, including depression and cognitive decline.^42–45^ Elevated systemic inflammatory mediator levels are thought to cause or contribute to depression by activating CNS inflammatory pathways and causing alterations in neurotransmitter function.^42,43^ Whether inflammatory pathways might mediate the relationship between diabetes and depressive symptoms bears further investigation.^46^ Furthermore, individuals with T1D are at greater risk for cognitive decline with aging compared to those without diabetes.^47^ Elevated levels of proinflammatory biomarkers in older adults have been linked to lower cognitive scores and more rapid declines in cognition.^48,49^ Characterizing how chronic inflammation might play a role in accelerating cognitive decline longitudinally in patients with T1D should be further investigated.

The current study has some limitations. Although we queried prospectively enrolled participants about any illnesses within the two weeks before study enrollment, we did not contact participants after enrollment to ensure that they did not become ill within a brief period after blood sample collection. Similarly, detailed information about illnesses within the two weeks preceding blood sample collection for the biobank samples was not available. We reviewed medical records for any diagnoses of other autoimmune or inflammatory conditions but did not conduct additional testing to rule out these disorders. It is therefore possible that some participants may have had undetected conditions affecting inflammation that could have increased variability in inflammatory mediator measurements, making associations with HbA1c more difficult to identify. In addition, data about other factors that are known to affect inflammation, such as diet and exercise, were not available for study participants. Differences in these factors may have caused additional variation in inflammatory mediator levels, obscuring some associations with HbA1c. Finally, continuous glucose monitoring data were not analyzed because these data were not available for biobank samples which represented more than half of the samples studied. Aspects of glycemia, such as glycemic variability, that are not reflected in HbA1c therefore could not be analyzed in this study.

In summary, the current study is the first to provide a broad cross-sectional characterization, across multiple inflammatory mediator classes, of the inflammatory profile resulting from hyperglycemia in children with uncomplicated T1D. We found that levels of multiple cytokines, chemokines, growth factors and MMPs were significantly correlated with HbA1c in this population. Among the inflammatory mediators most strongly correlated with HbA1c, several have known associations with microvascular complication of diabetes in adults, particularly IL-1β, IL-1RA, IL-6, TNF-α, VEGF, and CCL2. Longitudinal studies measuring inflammatory trajectories and monitoring for the development of complications would broaden our understanding of the involvement of systemic inflammation in causing T1D complications.

## Figures and Tables

**Table 1:** Participant characteristics (*n* = 117), mean (SD) or percent.

Age (years)	14.4 (2.9)
Sex (% female)	56%
Race^a^	
White	85%
Black	7%
Asian	3%
Other	5%
BMI (kg/m^2^)	22.9 (4.6)
Duration of diabetes (years)	7.4 (3.5)
Insulin regimen	
Multiple daily injections	28%
Insulin pump without AID	32%
Insulin pump with AID	38%
Unknown^b^	2%
HbA1c at time of inflammatory mediator measurement (%)	8.8 (1.7)

a Race data were missing for 3 participants. Ethnicity data were not recorded for the majority of biobank samples and are therefore not reported here.

b Insulin regimen information was unavailable for 2 participants who contributed samples to the biobank.

## Glossary

T1D: type 1 diabetes
HbA1c: hemoglobin A1c
DKD: diabetic kidney disease
BMI: body mass index
IL: interleukin
CCL: chemokine C–C motif ligand
CXCL: chemokine C-X-C motif ligand
MMP: matrix metalloproteinase
TNF: tumor necrosis factor
TRAIL: tumor necrosis factor related apoptosis inducing ligand
VEGF: vascular endothelial growth factor
TGF: transforming growth factor
PDGF: platelet derived growth factor
TPO: thrombopoietin
